# Access to General Practitioners during the second year of the COVID-19 pandemic in Portugal: a nationwide survey of doctors

**DOI:** 10.1186/s12875-023-01994-x

**Published:** 2023-02-13

**Authors:** Mónica Granja, Sofia Correia, Luís Alves

**Affiliations:** 1 EPIUnit - Instituto de Saúde Pública, Universidade do Porto, Rua das Taipas, N° 135, 4050-600 Porto, Portugal; 2 Laboratório para a Investigação Integrativa e Translacional em Saúde Populacional (ITR), Universidade do Porto, Rua das Taipas, N° 135, 4050-600 Porto, Portugal

**Keywords:** General Practitioners, Health services accessibility, Telemedicine, Primary health care, General practice

## Abstract

**Background:**

The Portuguese National Health System (NHS) provides universal coverage and near-free health care, but the population has high out-of-pocket expenses and unmet care needs. This suggests impaired accessibility, a key dimension of primary care. The COVID-19 pandemic has further affected access to health care. Understanding General Practitioners’ (GP) experiences during the pandemic is necessary to reconfigure post-pandemic service delivery and to plan for future emergencies. This study aimed to assess accessibility to GPs, from their perspective, evaluating determinants of accessibility during the second pandemic year in Portugal.

**Methods:**

All GPs working in NHS Family Practices in continental Portugal were invited to participate in a survey in 2021. A structured online self-administered anonymous questionnaire was used. Accessibility was assessed through waiting times for consultations and remote contacts and provision of remote access. NHS standards were used to assess waiting times. Descriptive statistics were used to characterize the study sample. Associations between categorical variables were tested using the χ2 statistic and the Student t-test was used to compare means of continuous variables.

**Results:**

A total of 420 GPs were included (7% of the target population). Median weekly working hours was 49.0 h (interquartile range 42.0–56.8), although only 14% reported a contracted weekly schedule over 40 h. Access to in-person consultations and remote contacts was reported by most GPs to occur within NHS time standards. Younger GPs more often reported waiting times over these standards. Most GPs considered that they do not have enough time for non-urgent consultations or for remote contacts with patients.

**Conclusions:**

Most GPs reported compliance with standards for waiting times for most in-person consultations and remote contacts, but they do so at the expense of work overload. A persistent excess of regular and unpaid working hours by GPs needs confirmation. If unpaid overtime is necessary to meet the regular demands of work, then workload and specific allocated tasks warrant review. Future research should focus on younger GPs, as they seem vulnerable to restricted accessibility. GPs’ preferences for more in-person care than was feasible during the pandemic must be considered when planning for the post-pandemic reconfiguration of service delivery.

**Supplementary Information:**

The online version contains supplementary material available at 10.1186/s12875-023-01994-x.

## Background

In primary care-based health systems, General Practitioners (GPs) are a common point of entry to the system for patients when a new health problem arises [1]. The availability of GPs has been found to correlate with better population health [2]. Thus, accessibility to GPs is a key feature of primary care-based systems [3]. The COVID-19 pandemic has affected access to health care. Staff was reduced due to infection and mandatory isolation [4, 5]. In-person consultations were restricted to prevent virus spread and telehealth was boosted [4, 6]. GPs were diverted to COVID-19-related work, further reducing accessibility in primary care [5].

The Portuguese health system is primary care-based, providing universal and mostly free coverage for health care (Table 1).

**Table 1:** Overview of the Portuguese health system [7–11]

The Portuguese health care system consists of several coexisting and overlapping systems: the universal NHS, health subsystems for certain professions, private health insurance, and the private sector
Coverage in the NHS includes most health services, except for dental care. Care is free in primary care, oncology and mental health services. The remaining services charge low user fees but only to adults with an income above a defined amount
Maximum waiting times for services are set by the government but compliance is uneven across both primary and secondary care
GPs are largely salaried public servants working in approximately 920 group Family Practices owned by the NHS and grouped into 55 Health Centres Groups
In NHS Family Practices, each GP cares for a list of about 1700 patients but over 10% of the population has no assigned GP due to a growing shortage of GPs working for the NHS
Family Practices in the NHS are organized in three models:
• 32% are Model B clinics, using a mix of salaried, capitation and pay-for-performance schemes
• 33% are Model A clinics, using a salary-only scheme while organizing their practices to reach model B status
• 35% are ‘UCSP’ clinics, using a salary-only scheme without the intention of reaching model B and having the additional task of caring for patients without an assigned GP
Telephone contact between GPs and their patients is a common practice but, before the pandemic, less than half of GPs reported using electronic mail with their patients on a regular basis
The NHS patient internet portal, launched in 2013, includes online GP appointment booking, repeat prescription ordering and a personal health record function, where patients can enter their health data

*NHS* National Health Service, *GP* General Practitioner, *‘UCSP’* Personalized Health Care Units

In spite of universal coverage and near-free health care, Portugal ranks high in the OECD table regarding greater per capita out-of-pocket expenses and catastrophic health care spending [12]. In the pandemic year of 2020, it was the second worst country regarding unmet care needs [12]. Impaired access to care in the public sector may explain why patients choose to pay for private care. Non-compliance with maximum waiting times for in-person GP care was reported even before the pandemic [13]. Also, patient satisfaction with telephone access to GPs was lower than overall satisfaction with care [14].

Early in the pandemic, Portuguese National Health Service (NHS) Family Practices were directed to cancel non-urgent care and institute triage systems. Respiratory hubs were used to assess patients presenting with symptoms suggestive of COVID-19 [15]. Patients suspected as COVID-19 cases were entered into a national database [16]. Most infected patients were sent home to isolate and assigned to daily remote follow-up by their GP [17]. Thus, during the pandemic years of 2020 and 2021 and into early 2022, GPs were diverted from their work in Family Practices to the telephone follow-up of infected patients or to cover shifts in respiratory assessment hubs. From 2021 on, they were also assigned to work in vaccination centres. As a result, there was a substantial and sustained drop in in-person consultations with GPs [12].

Understanding how GPs have experienced accessibility during the pandemic is necessary to reconfigure post-pandemic service delivery and to plan for future health emergencies. This study aimed to assess accessibility to GPs in Portugal, including in-person and remote access, to describe available resources and the views and experiences of GPs, and to evaluate determinants of accessibility during the second year of the pandemic.

## Methods

This cross-sectional study used data from a survey of GPs working in NHS Family Practices in continental Portugal. It was part of a larger study on access to GPs which also included a patient survey. On 13/05/2021, the Portuguese Medical Board sent an e-mail to all registered GPs, inviting them to answer a structured online self-administered anonymous questionnaire. It used LimeSurvey software and required about 12 min to complete. The Medical Board files include the estimated target population of 5684 GPs working in the public sector in Portugal in May 2021 [18], as well as retired GPs and those working only in the private sector. Exclusion criteria were applied with 3 qualifier questions: retirement or leave for over 6 months, doctors not doing any work in NHS Family Practices in continental Portugal, or those without a patient list. A reminder was sent on 04/08/2021 and valid replies were accepted if received by 31/08/2021.

The questionnaire was constructed by the investigators, after a literature search for comparable studies and adaptation of relevant questions from retrieved questionnaires [19–28]. Four GPs and one statistician discussed the face and content validity of the questionnaire. A pilot study on a convenience sample of final year Family Medicine residents also informed the final version of the questionnaire (Supplementary file 1). The areas covered included: actual working hours (excluding paid overtime); allocation of time for in-person care (office and home visits), remote contacts (telephone consultations, patient e-mails, video consultations, renewals of prescriptions and medical reports), and non-clinical work (management, meetings, continuing medical education, student/resident training); and demographics, including contracted weekly schedule, list size and Family Practice organizational model. Accessibility to GPs was assessed querying about waiting times for in-person consultations and remote contacts, provision of remote access, verification of data entered by patients into their personal area on NHS internet portal, views on accessibility arrangements, and available physical resources. GPs were asked to respond regarding the 4 working weeks before receiving the questionnaire.

NHS standards for provision of service were used to assess waiting times [8]. In-person appointments with one’s GP should be provided on the same day they are requested by the patient for acute illness. Appointments for non-acute reasons were to be arranged within 15 working days. Requests for home visits were to be met within 24 h. Remote requests for renewals of prescriptions and for issuing medical reports were to be met within 3 working days (Table 2). Given that no time standards are set for remote review of test results, telephone calls or e-mail contacts, the researchers set a cut-off of 3 working days, in line with the standards for other remote contacts. Provision of remote access was classified as ‘restricted’ if reported to be made available for ‘some’, ‘a few’ or ‘none’ of the patients, and as ‘broad’ is available for either ‘all ‘ or ‘many’ patients.

**Table 2:** Assessment of waiting times for General Practitioner services

	**maximum waiting times**
urgent consultation	same day^a^
non-urgent consultation	15 working days^a^
home visit	24 hours^a^
prescription renewal	3 working days^a^
medical reports	3 working days^a^
review of test results	3 working days^b^
return phone call from GP	3 working days^b^
GP reply to e-mail	3 working days^b^

^a^set by the Portuguese government; ^b^chosen by the authors

Descriptive statistics were used to characterize the study sample and compare participants to the population of GPs working in Portugal [10, 29, 30]. Associations between accessibility (waiting times and provision of remote access) and GPs’ demographic and professional characteristics were sought. Associations between categorical variables were tested using the χ^2^ statistic and the Student t-test was used to compare means of continuous variables. A sensitivity analysis was conducted to assess if answering the questionnaire in the first half of the study period (as opposed to the second half) influenced the main outcomes (weekly working hours and compliance with NHS standards for waiting times).

The study protocol was approved by the Ethics Committee of Matosinhos Local Health Unit on 10/07/2020 (nr. 59/CE/JAS).

## Results

**Fig. 1:**
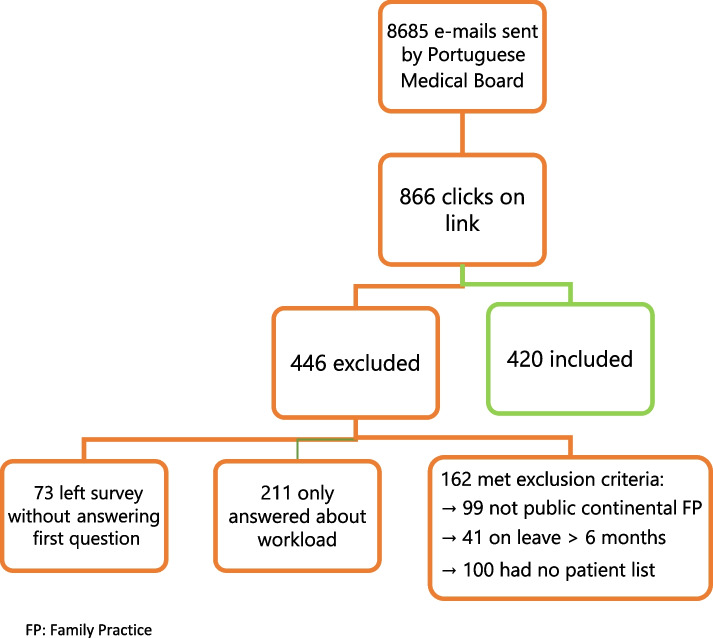
Enrolment of study participants. FP: Family Practice

The Medical Board sent e-mail invitations to 8685 doctors to participate in the study, including the estimated population of 5684 GPs working in the public sector in Portugal in May 2021 [18]. There were 866 clicks on the link to the questionnaire. Exclusion criteria applied to 162 answers. Questionnaires without answers to items about consultations were also excluded (*n* = 284). A total of 420 participants were included, representing 7% of the estimated total population and 60% of those who opened the questionnaire and were eligible (Fig. 1).

### Sample characterization

Most participants (68%) were female, their median age was 41 years (minimum 30, maximum 68, interquartile range 37–57 years). There were participants from each of the 55 Health Centres Groups across Portugal. Most GPs (42%) were from the Northern region. As for the organizational model of Family Practice, 46% of GPs worked in Model B, 33% in Model A and 21% in ‘UCSP’ clinics (Table 3). The average list size was 1726 patients (SD 241.4).

**Table 3:** Characterization of participant GPs and comparison to General Practitioners working in Portugal in 2021

	**participant GPs**	**all GPs** **Portugal (2021)** [10, 29, 30]
	**%**	
**age, years**		
** ≤ 35**	19.8	19.4
**36–45**	44.8	32.8
**46–55**	12.8	31.8
**56-65**^**a**^	22.6	31.6
**female**	68.4	69.5
**region**		
**North**	42.1	39.0
**LTV**	19.2	32.9
**Centre**	33.9	19.1
**Alentejo**	1.3	4.7
**Algarve**	3.5	4.3
**Family Practice type**		
**‘UCSP’**	20.6	35.1
**model A**	33.0	33.0
**model B**	46.4	31.9
**list size, mean (crude/weighted)**		
**‘UCSP’**	1601/2047	1520/2057
**model A**	1720/2167	1678/2187
**model B**	1786/2258	1800/2315

*GPs* General Practitioners, *LVT* Lisbon and Tagus valley, *‘UCSP’* Family Practices on a salaried-only scheme; Model A: Family Practices on a salaried-only scheme but working to reach pay-for-performance and capitation schemes; Model B: Family Practices on a mix of salaried, pay-for-performance and capitation schemes

^a^GPs over 65 years old were excluded from the comparison because national data were not comparable, as this age group includes all retired GPs, who were excluded from the study

Compared to the general population of GPs in Portugal, participants were younger. The Centre region was overrepresented, while Lisbon and the Tagus Valley region and the Alentejo region were underrepresented. Model B Family Practices were overrepresented, while ‘UCSP’ type clinics were underrepresented (Table 3).

### Working hours and tasks performed

Most GPs (68%) had a contracted workload in their NHS Family Practice between 36 and 40 h per week. While only 14% of GPs had a contracted weekly schedule over 40 h, most (80%) reported that, excluding paid overtime, they worked over 40 h a week in their NHS Family Practice (Table 4). Reported median weekly working hours was 49.0 h (interquartile range 42.0–56.8). The maximum weekly hours that could be reported on the survey form was 60, and this amount was reported by 74 participants (18%), but there were several comments at the end of the questionnaire stating that the weekly workload was over 60 h.

**Table 4:** Workload of study participants

**weekly hours in NHS Family Practice**	**contracted** (*n* = 373)	**worked** (*n* = 417)
	**%**	
≤ 30	4.0	3.1
31–35	13.9	2.6
36–40	68.1	13.9
> 40	13.9	80.3
**weekly hours, mean (*n* = 417)**		**average hours**
non-urgent in-person visits		18.9
remote clinical work		9.1
urgent in-person visits (except respiratory)		8.1
vaccination centre		4.0
covid follow-up calls		2.2
acute respiratory hubs		1.8
non-clinical work		2.2
home visits		1.3
other		0.9
		**%**
**nonclinical roles in NHS Family Practice (*n* = 375)**		77.6
train GP residents		50.9
tutor junior doctors/students		34.7
practice principal		22.4
other		11.5
**other paid jobs outside NHS Family Practice (*n* = 374)**		31.3
private/social sector		25.1
public hospital		3.5
university		2.9
other		2.4

*NHS* National Health Service, *GP* General Practice

The largest share of the participants’ workload in NHS Family Practices was for non-urgent, in-person visits (19 h weekly, on average). The second and third largest were for remote clinical work, and for urgent in-person visits, excluding visits for acute respiratory complaints (Table 4). COVID-19 related work (work in vaccination centres, follow-up calls, and shifts in acute respiratory hubs) accounted for an average of 8 h weekly.

Among participant GPs, 77% played other nonclinical roles in their practices or health centres groups, most often (51%) as trainers of GP residents (Table 4). Paid work outside NHS Family Practices was reported by 31% of participants, most often in the private and social sector (25%) and, less often, in NHS hospitals and in universities.

### Remote care

Most GPs reported that all the consultation rooms in NHS Family Practices had external landline telephone access (71%) and internet access (97%), but 74% stated that no video cameras were available. Nearly all GPs (99.5%) were provided with a work e-mail account and 58% had a work mobile phone.

Most GPs stated they provided their patients with access to discuss medical queries both by e-mail and through the practice telephone line, though not via their work or personal mobile phones, nor by video consultation (Table 5). Most GPs reported ‘never’ (54%) or ‘seldom’ (25%) consulting the information that patients entered on the NHS patient internet portal.

**Table 5:** Provision of remote access by participant General Practitioners

**remote contacts**	n	**restricted**	**broad**
		n (%)	
call GP on the practice telephone	395	96 (24.3)	299 (75.7)
call GP on his work mobile	388	349 (89.9)	39 (10.1)
call GP on his personal mobile	393	377 (95.9)	16 (4.1)
discuss a medical query on the phone	395	109 (27.6)	286 (72.4)
discuss a medical query by e-mail	395	59 (14.9)	336 (85.1)
get a video consultation	390	366 (93.8)	24 (6.2)

*GP* General Practitioner

### Accessibility to the General Practitioner

Access to in-person office consultations and remote contacts was reported by most GP to occur within maximum waiting times (MWT) or up to 3 working days where MWT were not defined (Table 6). Home visits were reported to exceed MWT by 93% of GPs, and remote review of test results, reported to exceed 3 working days by 51%. Regarding time in the waiting room, 85% of GPs stated their consultations usually began up to 15 min after the scheduled time.

**Table 6:** Waiting times according to compliance with National Health Service standards

waiting time	total n	maximum waiting time^a^		no response to request (%)
		**within (%)**	**over (%)**	
urgent consultation	419	75.2	24.8	-
non-urgent consultation	418	54.1	45.9	-
home visit	418	6.9	93.1	-
prescription renewal	392	85.2	14.8	-
medical reports	392	55.1	44.4	0.5
		**working days**		
		** ≤ 3 (%)**	** > 3 (%)**	
review of test results	392	47.7	51.0	1.3
return phone call from GP	393	90.3	6.9	2.8
GP reply to e-mail	394	70.1	27.2	2.8

*GP*: General Practitioner

^a^maximum waiting time as defined by the national Health Service standards [8]

### Doctor’s views on accessibility arrangements

Most participant GPs consider that they do not have enough time for non-urgent consultations nor for remote contacts (especially for e-mails and telephone calls) (Table 7). Most GPs consider remote contacts (except for video consultations and data entry in the NHS portal) to be useful for patient management.

**Table 7:** General Practitioners’ views on available time for contacts and on the usefulness of remote contacts

	time				helps patient management	
		not enough	neutral		agree	neutral
	n	%		n	%	
urgent consultations	420	36.7	8.6	-	-	-
non-urgent consultations	419	64.9	8.1	-	-	-
home visits	420	39.0	10.7	-	-	-
prescriptions renewal	392	54.6	5.6	393	75.8	10.7
medical reports	394	72.3	5.6	392	64.8	17.1
test results reviews	393	74.6	6.6	392	68.6	11.0
return patients’ phone calls	395	81.8	5.6	394	75.4	10.7
reply to patients’ e-mails	394	83.2	6.3	393	71.5	13.5
video consultations	384	76.8	18.2	386	33.4	39.6
check information patient portal	392	82.7	12.2	389	31.6	47.3

GPs were asked if they would change their use of telephone calls, e-mails, and video consultations in some situations (Table 8). If real time access to patient files was available, most GPs stated they would use e-mail and video consultations more often (64% and 50%, respectively). If it was included in their performance assessment, most GPs (52%) stated they would use e-mail more often.

**Table 8:** General Practitioners’ intentions to change the use of telephone calls, e-mails, and video consultations

	**% would use more / % neutral (n)**		
	**if real time record to file available**	**if included in performance assessment**	**if video camera available**
telephone calls	37.2/26.0 (393)	48.7/21.6 (394)	-
e-mail	64.3/15.8 (392)	51.5/23.5 (392)	-
video consultations	50.4/23.5 (387)	44.3/28.2 (386)	41.7/23.3 (386)
	**% would use less / % neutral (n)**		
	**if more time available for in-person consultations**		
telephone calls	69.4/12.4 (395)		
e-mail	57.6/17.4 (373)		
video consultations	62.0/28.2 (71)		

### Provision of telephone and e-mail access and GP characteristics

Younger GPs more often reported restricted telephone and e-mail access (Table 9). GPs from the Centre region more often reported restricted access to patients through the practice telephone line and mobile telephones provided at work. GPs working in the ‘UCSP’ model of Family Practices most often reported restricted access to the discussion of medical queries by e-mail, followed by those working in model A Family Practices. GPs working only in NHS Family Practices more often reported restricted access through their personal mobile phone. GPs working more hours more often reported broad access to discuss medical queries by e-mail.

**Table 9:** Restricted (versus broad) remote access according to characteristics of General Practitioners

GP characteristics	practice phone line	work mobile	personal mobile	phone medical query	medical query e-mail	video consultation
	**% restricted access**					
**sex**						
female	23.3	90.5	96.9	25.7	13.2	94.9
male	27.7	89.0	93.3	31.9	17.6	92.3
*p*-value	0.359	0.655	0.109	0.207	0.260	0.325
**age (years)**						
≤ 35	35.2	100	98.6	43.7	23.9	93.0
36–45	23.6	93.1	97.5	30.4	14.3	92.4
46–55	10.9	87.0	95.7	10.9	10.9	97.8
> 55	25.0	78.0	90.5	19.8	9.4	95.8
*p*-value	**0.028**	** < 0.001**	**0.044**	** < 0.001**	0.054	0.524
**region of practice**						
North	17.7	88.5	94.3	21.5	16.5	94.9
Centre	39.4	96.0	97.6	40.2	11.0	92.7
Other	16.7	85.4	95.5	21.1	16.7	95.5
*p*-value	** < 0.001**	**0.023**	0.371	** < 0.001**	0.361	0.711
**Family Practice model**						
‘UCSP’	23.4	87.0	96.1	32.5	29.9	93.5
model A	31.7	91.1	98.4	31.7	16.3	92.6
model B	20.2	91.0	93.6	22.5	6.9	95.3
*p*-value	0.075	0.573	0.133	0.125	** < 0.001**	0.621
**non-clinical roles**						
no	25.5	89.3	96.0	28.3	14.7	93.7
yes	14.3	95.1	95.2	21.4	16.7	95.1
*p*-value	0.109	0.407	0.684	0.344	0.739	1
**other paid work**						
no	24.1	90.1	97.7	29.2	14.8	95.7
yes	26.5	90.6	92.3	24.8	14.5	90.5
*p*-value	0.623	0.876	**0.014**	0.379	0.948	0.052
**contracted weekly workload (hours)**						
up to 35	22.4	87.9	100.0	26.9	14.9	95.5
36–40	26.4	90.4	94.5	30.7	15.4	93.7
> 40	21.2	92.2	96.2	15.4	11.5	94.0
*p*-value	0.634	0.718	0.120	0.079	0.778	0.944
	**broad versus restricted access**					
**crude list size**						
mean difference [95% IC]	-36 [-93, 21]	88 [5, 170]		21 [-34, 76]	-39 [-109, 31]	
*p*-value	0.215	**0.037**	non-normal	0.461	0.27	non-normal
**weighted list size**						
mean difference [95% IC]	-21 [-97, 54]	42 [-70, 154]		-3 [-75, 70]	-33 [-129, 63]	
*p*-value	0.573	0.464	non-normal	0.943	0.505	non-normal
**average weekly hours**						
mean difference [95% IC]	-1.0 [-3.1, 1.2]	-1.5 [-4.6, 1.6]	-0.3 [-4.9, 4.4]	0.1 [-2.2, 2.4]	2.8 [0.2, 5.4]	[-4.5, 3.2]
*p*-value	0.374	0.346	0.909	0.953	**0.032**	0.745

Significant *p*-values are shown in bold

*GP* General Practitioner, *LVT* Lisbon and Tagus valley, *‘UCSP’* Family Practices on a salaried-only scheme; Model A: Family Practices on a salaried-only scheme but working to reach pay-for-performance and capitation schemes; Model B: Family Practices on a mix of salaried, pay-for-performance and capitation schemes

### Waiting times and GP characteristics

The age of the GP was associated with waiting times. Younger participants more often reported waiting times over MWT for non-urgent consultations and for all types of remote contacts (or over 3 working days where MWT were not defined) (Table 10). GPs working in the North region, followed by those from the Centre region, more often reported waiting times over MWT for non-urgent consultations. GPs working in the Centre more often reported waiting times over 3 working days for remote medical reports, telephone, and e-mail contacts. GPs working in practices on a salary-only scheme (‘UCSP’), followed by GPs working in model A Family Practices, more often reported waiting times over MWT for non-urgent consultations. The only factor associated with longer waiting times for urgent consultations was the contracted weekly workload. GPs with less contracted hours more often reported waiting times over MWT. GPs with smaller lists more often reported waiting times over MWT for requests for remote medical reports and for review of test results. Total weekly hours worked were not associated with waiting times (Table 10).

**Table 10:** Waiting times over the National Health Service standards, according to characteristics of General Practitioners

	**% over maximum waiting time**^**a**^						**% over 3 working days**		
**GP characteristics**	**urgent consultation**		**non urgent consultation**	**home visit**	**prescription renewal**	**medical report**	**telephone call with GP**	**e-mail with GP**	**test results review**
**sex**									
female	24.1		48.0	92.6	14.5	45.5	6.9	29.4	54.2
male	28.0		37.8	94.1	15.1	41.5	7.8	23.4	40.2
*p*-value	0.427		0.064	0.605	0.864	0.473	0.755	0.260	0.012
**age (years)**									
≤ 35	31.0		65.7	95.7	22.5	53.5	17.6	50.0	65.2
36–45	21.7		50.3	96.3	16.9	47.5	7.1	27.7	56.3
46–55	26.1		50.0	80.4	13.0	47.8	2.2	20.0	50.0
> 55	26.3		17.7	91.7	6.3	29.8	2.2	14.1	28.0
*p*-value	0.500		** < 0.001**	**0.005**	**0.022**	**0.010**	**0.002**	** < 0.001**	** < 0.001**
**region of practice**									
North	27.8		51.9	91.7	12.0	43.0	3.9	21.7	49.0
Centre	25.4		45.7	93.7	15.1	53.6	13.9	38.9	54.0
Other	21.1		31.5	94.4	18.9	32.6	3.4	21.8	45.5
*p*-value	0.503		**0.008**	0.676	0.337	**0.009**	**0.003**	**0.002**	0.452
**Family Practice model**									
‘UCSP’	35.1		53.2	94.8	15.6	45.5	5.6	23.3	49.3
model A	22.8		49.6	95.9	20.5	50.8	8.3	28.9	58.3
model B	22.1		37.8	90.2	10.4	39.3	6.5	28.4	44.2
*p*-value	0.072		**0.034**	0.133	0.054	0.145	0.766	0.657	0.059
**nonclinical roles**									
no	24.9		46.0	92.8	13.7	44.5	6.8	28.1	50.4
yes	23.8		45.2	95.2	23.8	45.2	9.5	26.8	61.9
*p*-value	0.873		0.924	0.755	0.082	0.932	0.520	0.867	0.160
**other paid work**									
no	22.7		45.3	93.8	13.6	44.3	7.2	31.2	50.8
yes	30.8		44.4	92.2	17.2	44.0	7.1	20.2	48.2
*p*-value	0.094		0.876	0.584	0.361	0.950	0.959	**0.029**	0.652
	**% over maximum waiting time**						**% over 3 working days**		
**GP characteristics**	**urgent consultation**		**non urgent consultation**	**home visit**	**prescription renewal**	**medical report**	**telephone call with GP**	**e-mail with GP**	**review test results**
**contracted weekly workload (hours)**									
up to 35		35.8	41.8	97.0	10.4	49.3	6.1	27.3	54.5
36–40		24.8	47.4	92.5	17.0	43.1	7.7	29.1	49.0
> 40		15.7	36.5	90.4	9.6	42.0	6.1	22.0	48.0
*p*-value		**0.041**	0.303	0.286	0.214	0.632	0.952	0.585	0.697
		**within versus over maximum waiting time**					**within versus over 3 working days**		
**crude list size**									
mean difference [95% IC]		29 [-28, 86]	20 [-29, 70]		76 [-11, 164]	83 [32, 135]		58 [2, 113]	72 [23, 121]
*p*-value		0.316	0.421	non-normal	0.086	**0.002**	non-normal	0.041	**0.004**
**weighted list size**									
mean difference [95% IC]		45 [-31, 121]	18 [-48, 83]		-9 [-98, 79]	51 [-158, 116]		45 [-30, 120]	40 [-25, 105]
*p*-value		0.242	0.597	non-normal	0.833	0.128	non-normal	0.236	0.231
**average weekly hours**									
mean difference [95% IC]		-0.5 [-2.6, 1.6]	-0.6 [-2.4, 1.2]		0.5 [-2.1, 3.1]	0.4 [-1.5, 2.2]	-0.2 [-3.9, 3.4]	-0.4 [-2.5, 1.6]	0.2 [-1.6, 2.1]
*p*-value		0.61	0.522	non-normal	0.683	0.685	0.901^a^	0.672	0.818

Significant *p*-values are shown in bold; ^a^maximum waiting time as defined by the national Health Service standards [8]

*GP* General Practitioner, *LVT* Lisbon and Tagus valley, *‘UCSP’* Family Practices on a salaried-only scheme; Model A: Family Practices on a salaried-only scheme but working to reach pay-for-performance and capitation schemes; Model B: Family Practices on a mix of salaried, pay-for-performance and capitation schemes

### Sensitivity analysis

The results of the sensitivity analysis to assess the impact of the time the questionnaire was answered were similar to those based on primary analysis.

## Discussion

In this study, the main finding is that most GPs report compliance with standards for waiting times for in-person office consultations and remote contacts, but they do so at the expense of work overload.

GPs reported working on average 49 h a week in Family Practices of the Portuguese NHS, excluding paid overtime. Many GPs reported regularly working 60 or more hours per week. This is substantially more than the findings of previous research [31, 32] and exceeds the maximum of 40 regular weekly hours mandated by Portuguese law. Weekly hours reported by GPs across European countries has ranged from 33 to 51 h [32]. Lately, both increasing and decreasing trends were found in GP working hours in Europe [33, 34]. Excessive working hours may be related to extra tasks assigned to GPs during the pandemic, with no change in traditional tasks. Other explanations may be unrelated to the pandemic. The demand for health care has been growing due to increasing list size, aging of the population, growing medical complexity of patients, and increased work by GPs due to long waiting times for hospital appointments. Also, excessive bureaucracy in Portugal leads to the request of many medical reports and certificates that are not health care driven. Excessive workload should be a concern because it is one of the factors leading to professional burnout [35, 36], and adverse patient outcomes [37]. Work overload is also associated with intention to leave the profession [38]. The shortage of GPs working for the NHS is growing in Portugal, as in other primary care-based systems [39]. Despite having one of the highest ratio of GPs to inhabitants in OECD countries [12], GPs in Portugal are increasingly choosing to leave the public sector, increasing the proportion of the population with no assigned GP, which is currently over 10% [10]. Reduction in list sizes has been a recurring demand from Portuguese GP unions and professional associations [40]. Freeing GPs from excessive bureaucracy-driven work and from low value care may also control workload and improve productivity, and health outcomes [41, 42]. Some degree of task shifting may be necessary, provided it is informed by research, in order to keep the benefits of the discipline [43, 44].

In-person office consultations accounted for the largest share of the GPs workload, followed by remote care and by COVID-19 related work. Previous research in Portugal found that in-person visits occupied a bigger share of the workload, but in less hours per day [31]. Thereafter, the increase in total hours found in our study has come at the expense of both in-person and remote contacts.

Portuguese Family Practices were reported by participants to be well equipped regarding internet access, but not so regarding telephone equipment and even less so for video consultations. The pandemic has strained telephone access to primary care [45] and it was seen as an opportunity for improvement [46]. However, GPs stated they lacked the time for remote contacts and would not be willing to increase telephone contacts, even if systems improved or it was valued in appraisal. Furthermore, varying informatic literacy among different population groups may modulate these findings.

Waiting times for in-person office consultations and for most remote contacts were most often reported to comply with maximum waiting times (MWT) or 3 working days (if MWT were not defined). This findings need confirmation given that non-compliance has been reported [9, 13]. However, most GPs consider they do not have enough time for non-urgent consultations or for remote contacts, even though they find the latter to be helpful. The mean consultation length in Portuguese Family Practices has been estimated as 16 min [31], one of the longest in the world [47]. However, the trade-off between consultation length and the number of consultations available may be hard to manage. Lack of time for tasks considered to be important exacerbates overload. Home visits were the only contact most often reported to have waiting times longer than the MWT of 24 h. Given that most GPs considered they had enough time for home visits, this MWT may need review. Access for the discussion of questions from patients, both by the practice phone line and by e-mail access, was also reported by most GPs. However, most GPs stated they would do fewer remote contacts if they had enough time for in-person consultations. GPs may be using remote contacts to help them cope with increasing demand. Remote care is better accepted by GPs and their patients when there is an established doctor-patient relationship [48]. This ongoing relationship requires nurturing. Research has highlighted accessibility to GPs as inextricable from continuity [49]. Moreover, to prevent widening the digital divide, remote access must be kept as an add-on and traditional channels must be preserved [50, 51]. Remote care cannot limitlessly replace in-person care, solely in the interest of coping with demand [52].

Young GPs were more likely to report non-compliance with MWT. Non-compliance was not associated with list size, Family Practice organizational model, or total working hours. Younger age was also associated with provision of more restricted access to telephone and e-mail contacts. Early in their careers, GPs are more exposed to time stress [53]. This may be related to being less experienced in managing workload or because it takes time to get to know patients and establish therapeutic relationships. Moreover, shorter duration of relationships between young GPs and their patients may lead to less acceptance of remote care [48]. When remote access is restricted, it may be more difficult to manage demand. It is unlikely that the composition of the lists of younger doctors can explain longer waiting times. Younger doctors do not select lists of younger patients but most often inherit the list of an older doctor who retires or of a colleague who leaves the practice. Practice lists tend to be similar within a given Family Practice in terms of age, gender, and health problems presented. Beyond the activities mandated by quality indicators in pay-for-performance schemes, additional procedures such as minor surgery or home visits may characterize individual practices. A future study of the task profile of GPs in Portugal by age would be helpful in clarifying this.

Other GP factors were associated with waiting times and with the provision of remote access but in a less consistent pattern. GPs working in the North region most often reported non-compliance with MWT for non-urgent consultations. Consumer surveys and monitoring of MWT in Portuguese Family Practices confirms higher rates of non-compliance with waiting times for non-urgent consultations in the North region [9, 13]. These findings warrant reflexion, as the North region is where the shortage of GPs is less of a problem. GPs from the Centre region most often reported requiring more than 3 working days to return a phone call or reply to an e-mail, or to provide a remote medical report, and more often reported provision of restricted access to telephone contacts. This may be explained by a local organizational culture trading off in-person consultations against remote care.

GPs working in Model B Family Practices, followed by those working in Model A, less often reported waiting times over MWT for non-urgent consultations, and less restrictions on e-mail access. This may be because waiting times are part of the quality framework in place in pay-for-performance schemes of Model B Family Practices. On the other hand, GPs in ‘UCSP’ clinics have the additional task of caring for patients with no assigned GP, rendering them less available to their own patients.

GPs reporting waiting times up to 3 working days to respond to remote requests for medical reports and to review test results had larger list sizes than those who reported requiring more than 3 working days. GPs providing broad access through work mobile telephones and e-mail, had average larger list sizes. This may mean that, to cope with demand, GPs with larger lists are more prone to provide faster and broader remote access.

Questions about video consultations and access to patient information on the NHS patient portal (launched in 2013) got the highest proportion of ‘never used’ and neutral views, suggesting slow adoption by GPs. In the case of video consultations, this may be due to the lack of perceived benefit [54] or to the restricted availability of video equipment. Limited use of the patients’ NHS internet portal by GPs may be related to lack of perceived benefit, work overload, or online fatigue [55].

### Strengths and limitations

To our knowledge, this is the first study in Portugal exploring GPs’ resources, experiences, and views on accessibility to their care, considering both in-person and remote contacts. The pandemic made it even more relevant to address accessibility, due to the restrictions to in-person access and to the expansion of remote access. A patient survey was conducted simultaneously to achieve a comprehensive perspective.

The study has some limitations. First, the questionnaire used has not been validated. However, it was based on validated, published questionnaires assessing accessibility. It has been subjected to face and content validation and a pilot study informed its final version.

Second, the response rate was low, limiting generalizability of results. GPs were recruited as a census but less than 10% of recipients of the invitation to participate clicked on the link to the questionnaire. However, 60% of those who opened the questionnaire answered it and there were participants from every Health Centres Group in continental Portugal. To maximize the response rate, an e-mail reminder was sent, appeals for participation were made on social media, and the study period was expanded. Participants were younger than the population of GPs working in Portugal, and GPs working in the Centre region and Model B Family Practices were over-represented. Younger GPs, lacking the experience of their older colleagues, may require more time for consultations and hence generate longer waiting times for appointments. In model B clinics, the pay-for-performance scheme rewards compliance with maximum waiting times, but the demands of keeping up with all the standards of quality indicators may also result in longer waiting times for patients. GPs training Family Medicine residents were also over-represented in this study as around 20% of GPs are estimated to be trainers. Training may affect waiting times in opposite ways: the time demands of training may increase waits, but the extra work force of residents may decrease them. The low response rate may be partly explained by the increase in workload GPs are experiencing, and by online fatigue [55].

Third, survey studies are prone to information biases. Asking questions to the past 4 weeks of practice sought to minimize recall bias when answering about usual practices. Self-report of working hours and compliance with standards for waiting times may be over-estimated, but the possibility of under-estimation cannot be ruled out either [56]. The limit of 60 to the maximum weekly hours that could be reported may have decreased the median weekly working hours as it was criticised by several participants who reported to work over 60 h a week. Under-reporting bias may arise in questions addressing practices (like waiting times) that, even with anonymity, may produce an undesirable collective picture. GPs struggling to comply with MWT may find it harder to participate in research [57]. The results of the patient survey on accessibility to GPs that was launched simultaneously will shed light on these findings.

## Conclusions

A persistent excess of regular and unpaid working hours by GPs needs confirmation and monitoring. If unpaid overtime is persistently necessary to meet the regular demands of work, then workload in general and specific allocated tasks warrant review. Reduction in list sizes and freeing GPs from excessive bureaucracy-driven work and from low value care need to be considered. GPs’ preferences for more in-person care than was feasible during the pandemic must be considered when planning for the post pandemic reconfiguration of service delivery. Future research should focus on younger GPs, as they seem especially vulnerable to restricted accessibility. Clarifying if the age differences found are generational (possibly related to a stronger drive to secure work-life balance), or an attribute of early career GPs (lacking clinical experience, in the process of building relationships with their patients, or experiencing childcare challenges), could allow for targeted interventions.

### Supplementary Information

**Additional file 1.**

## Data Availability

The datasets used and analyzed during the current study are available from the corresponding author on request.

## Abbreviations

GP: General Practitioners
MWT: Maximum waiting times
NHS: National Health Service
