# Correction: Access to General Practitioners during the second year of the COVID‑19 pandemic in Portugal: a nationwide survey of doctors

**DOI:** 10.1186/s12875-023-02220-4

**Published:** 2023-12-01

**Authors:** Mónica Granja, Sofia Correia, Luís Alves

**Affiliations:** 1 EPIUnit – Instituto de Saúde Pública, Universidade do Porto, Rua das Taipas, N° 135, 4050‑600, Porto, Portugal; 2 Laboratório para a Investigação Integrativa e Translacional em Saúde Populacional (ITR), Universidade do Porto, Rua das Taipas, N° 135, 4050‑600, Porto, Portugal

**Correction: BMC Primary Care 24:46 (2023)**

10.1186/s12875-023-01994-x

Following publication of the original article [1], the author reported that author names for reference 32 is incorrect. It should be corrected to “Schäfer WLA, Van Den Berg MJ, Groenewegen PP”.
