# Supplementary material for: Access to General Practitioners during the second year of the COVID-19 pandemic in Portugal: a nationwide survey of doctors
Source: BMC Prim Care. 2023 Feb 13;24:46. doi: 10.1186/s12875-023-01994-x (PMC9924884; doi:10.1186/s12875-023-01994-x)
Supplement: Supplementary file 1 — Additional file 1. [file 12875_2023_1994_MOESM1_ESM.pdf]

SUPPLEMENTARY FILE 1
STUDY QUESTIONNAIRE

Accessibility to the General Practitioner
– a comprehensive approach during the pandemic

This survey aims at understanding how you organize and perceive your patients' accessibility to your care. It focuses on remote access with particular detail, as they were boosted by the covid-19 pandemic. Answering the survey is voluntary and anonymous, taking about 12 minutes. This research is nested in a PhD Thesis that will query patients as well [you can find further information here – link to the study information leaflet]. The study protocol was approved by Matosinhos Local Health Unit Ethical Review Board.

We ask you to answer considering what happened most often during the past 4 weeks, excluding any period of annual leave. It is important that you answer as accurately as possible.

Any queries about the study or its results should be addressed to the research team: Mónica Granja (General Practitioner), Luís Alves, PhD (General Practitioner) e Sofia Correia, PhD (researcher), xxx@med.up.pt or 91xxx.

1. **Do you work as a General Practitioner in a Family Practice in continental Portugal?**
    - no [exclusion criteria]
    - yes, and currently I am working or on leave for less than 1 month
    - yes, but currently I am on leave for over 1 month [exclusion criteria]

2. **Are you responsible for a list of patients?**
    - no [exclusion criteria]
    - yes

3. **Excluding paid overtime, for the last 4 weeks, how many hours did you spend weekly on…**
    - urgent consultations (excluding covid assessment hubs)?
    - non-urgent consultations?
    - covid assessment hubs?
    - vaccination centres?
    - home visits?
    - remote clinical work, excluding covid follow-up calls (prescription renewals, phone calls and e-mails with patients, review of test results, reports, records, care coordination with relatives, carers or other health staff/referrals)?
    - covid follow-up calls?
    - non-clinical work (meetings, continuing medical education, student/resident training)?
    - other activities?

about in-person consultations

In some instances, it is not possible to comply with maximum waiting times. We ask you to answer considering what happened most often for the past 4 weeks, excluding any period of annual leave.

4. **When a patient from your list needs an URGENT CONSULTATION, does he usually get it…**
    - on the same day?
    - in 1 or 2 working days?

- in 3 or 4 working days?
- in 5 working days or more?

5. **When a patient from your list needs a NON-URGENT CONSULTATION, does he usually get it…** (in up to 1 week? in 2 to 3 weeks? in 4 to 8 weeks? in 9 to 12 weeks? in 3 months or more?)
   - general adult consultation
   - child care
   - antenatal care
   - family planning
   - diabetes management
   - hypertension management

6. **When a patient from your list needs a HOME VISIT, does he usually get it…**
   - in 1 working day?
   - in 2 or 3 working days?
   - in 4 or 5 working days?
   - in more than 5 working days?

7. **Usually, when do your appointments start, in relation to the scheduled time?**
   - in time, or even before that
   - in up to 15 minutes
   - in 16 to 30 minutes
   - in 31 to 60 minutes
   - in more than 60 minutes

8. **How much do you agree or disagree with the following statements?** (strongly disagree, somewhat disagree, neither agree nor disagree, somewhat agree, strongly agree)
   - I have enough appointment slots for URGENT CONSULTATIONS
   - I have enough appointment slots for NON-URGENT CONSULTATIONS
   - I have enough appointment slots for HOME VISITS
   - It is up to me to decide the length of each appointment slot
   - It is up to me to decide the ratio of slots for URGENT, NON-URGENT and HOME VISITS

about remote clinical work

Remote clinical work is considered as any task in relation to a specific patient who is not present (prescription renewals, phone calls and e-mails with patients, review of test results, reports, records, care coordination with relatives, carers or other health staff/referrals)**.**

Please consider what happened most often for the past 4 weeks, excluding any period of annual leave.

9. **In relation to the consultation rooms where you work as a GP, which option best describes them?**
   - none of them has a landline
   - some have a landline, but you cannot make external calls yourself
   - some have a landline from which you can make external calls
   - all of them have a landline from which you can make external calls

10. **Do you have a work mobile phone provided by your organization?**
    - no
    - yes

11. May the patients from your list… (all, many, some, a few, none)

- call you through the practice phone line?
- call you on your work mobile phone?
- call you on your personal mobile phone?

12. **May the patients from your list call you on the phone with a medical query?**

- all
- many
- some
- a few
- none

13. If a patient has tried to call you but could not reach you and left a message requesting you a phone call, usually how long will he wait for you to return his call?

- up to 1 working day
- 2 or 3 working days
- 4 or 5 working days?
- more than 5 working days
- usually, I don't return patient calls

14. **Over the past 4 weeks how often did you call your patients to monitor their health condition in between appointments?**

- never
- seldom
- often
- very often

15. **How much do you agree or disagree with the following statements?** (strongly disagree, somewhat disagree, neither agree nor disagree, somewhat agree, strongly agree)

- I have enough time to answer/return patient calls
- I would use the telephone with my patients more often if I could record calls into patients' files in real time
- I would use the telephone with my patients more often if calls were accounted for in performance evaluation
- phone calls with patients improve the management of patients and appointments
- I would use the telephone with my patients less often if I had enough time for in-person appointments

16. **Do the consultation rooms where you work as a GP** (considering both in-person and remote work) **have a computer with internet access?**

- yes, some of them
- yes, all of them

17. Do you have a work e-mail address?

- no
- yes
- don't know / not sure

18. **May the patients from your list send you an e-mail with a medical query?**
    - all
    - many
    - some
    - a few
    - none

19. **Usually, usually how long will a patient wait for you to reply to an e-mail?**
    - up to 1 working day
    - 2 or 3 working days
    - 4 or 5 working days?
    - more than 5 working days
    - usually, I don't reply to patient e-mails

20. **Excluding covid follow-up contacts, how often do you e-mail your patients to monitor their clinical condition in between appointments?**
    - never
    - seldom
    - often
    - very often

21. **Do you check the data that your patients enter into their ‘personal area/health summary’ on the National Health Service patient portal?**
    - never
    - seldom
    - sometimes
    - often
    - always

22. **How much do you agree or disagree with the following statements?** (strongly disagree, somewhat disagree, neither agree nor disagree, somewhat agree, strongly agree)
    - I have enough time to read/reply to patient e-mails
    - I have enough time to check the data that patients enter into their ‘personal area/health summary’ on the National Health Service patient portal
    - I would use e-mail with my patients more often if messages could be automatically recorded into their files
    - I would use e-mail with my patients more often if that was accounted for in performance evaluation
    - e-mail with patients improves the management of patients and appointments
    - checking the data that patients enter into their ‘personal area/health summary’ improves the management of patients and appointments
    - I would use e-mail with patients less often if I had enough time for in-person appointments

23. **Are the computers in the consultation rooms where you work as a GP** (considering both in-person and remote work) **equipped for video consultations?**
- no
- yes, some of them
- yes, all of them
- don’t know / not sure

24. **May your patients get a video consultation with you at their request?**
- all
- many
- some
- a few
- none

25. **How much do you agree or disagree with the following statements?** (strongly disagree, somewhat disagree, neither agree nor disagree, somewhat agree, strongly agree)
- I have enough time for video consultations
- I would do more video consultations if the computers I use were equipped for video consultations
- I would do more video consultations if they could be booked and recorded into patients’ files
- I would do more video consultations if they were accounted for in performance evaluation
- video consultations improve the management of patients and appointments
- I would do fewer video consultations if I had enough time for in-person appointments

26. In the following question one acknowledges that there are situations in which one cannot comply with maximum waiting times. Most often, for the past 4 weeks, how long will a patient wait if he requests a remote… (up to 1 working day, 2 or 3 working days, 4 or 5 working days, more than 5 working days, usually I don't do it)
- prescription renewal?
- medical report?
- review of test results?

27. How much do you agree or disagree with the following statements? (strongly disagree, somewhat disagree, neither agree nor disagree, somewhat agree, strongly agree)
- I have enough time for remote prescription renewals
- I have enough time for remote medical reports
- I have enough time for remote review of test results
- remote prescription renewals improve the management of patients and appointments
- remote medical reports improve the management of patients and appointments
- remote review of test results improves the management of patients and appointments
- It is up to me to decide the available time slots for remote contacts

28. What is your opinion about the following aspects of your practice for the past 4 weeks?
(1 = poor/2/3/4/5 = excellent/not applicable/not relevant)
- easiness for patients to book a suitable appointment?
- easiness for patients to get through to the practice on the phone?
- easiness for patients to get through to you on the phone?
- time in the waiting room?
- quickness with which urgent problems get sorted?

29. How do you compare the following aspects of your practice before and after the pandemic?
(much worse before/worse before/no difference/better before/much better before)

- easiness for patients to book a suitable appointment?
- easiness for patients to get through to the practice on the phone?
- easiness for patients to get through to you on the phone?
- time in the waiting room?
- quickness with which urgent problems get sorted?

about you

How old are you? ________

What is your gender?

- female
- male

Which region do you work in? (North, Centre, Lisbon and Taggus Valley, Alentejo, Algarve)

Which Health Centres Group do you work in?

Which model of Family Practice do you work in?

- UCSP
- Type A
- Type B

How many weekly working hours does your contract state?

- up to 20
- 21-30
- 31-35
- 36-40
- 41 and over

What is the approximate size of your patient list?

- crude size _________ patients
- weighted size _________ patients

Do you do other paid work apart from your National Health Service Family Practice?

- no
- yes

If you do, where is it? (you can choose any number of applicable options)

- public sector hospital
- private or social health sector hospital or clinics
- other, which?

30. **Over the past 4 weeks did you take any of the following roles in your National Health Service Family Practice/Health Centres Group?** (you can choose any number of applicable options)

- president of clinical committee of Health Centres Groups
- practice principal
- trainer of Family Medicine residents
- trainer of other residents
- trainer of Medicine students
- other, which?
